# In Silico Analysis of C_60_ Fullerene Interaction with TMPRSS2: Toward Novel COVID-19 Prevention Approaches

**DOI:** 10.3390/molecules30234586

**Published:** 2025-11-28

**Authors:** Vasyl Hurmach, Viacheslav Karaushu, Svitlana Prylutska, Zinaida Klestova, Sergiy Vyzhva, Yuriy Prylutskyy, Uwe Ritter, Vasil Garamus

**Affiliations:** 1 Institute of Molecular Biology and Genetics, National Academy of Science (NAS) of Ukraine, 03143 Kyiv, Ukraine; vhurmach@gmail.com; 2 Taras Shevchenko National University of Kyiv, 01601 Kyiv, Ukraine; karaushu@gmail.com (V.K.); s.vyzhva@knu.ua (S.V.); prylut@ukr.net (Y.P.); 3 National University of Life and Environmental Science of Ukraine, 03041 Kyiv, Ukraine; psvit_1977@ukr.net; 4 The Institute for Medical Virology and Epidemiology of Viral Diseases, 72076 Tübingen, Germany; zinaklestova@gmail.com; 5 Technical University of Ilmenau, 98693 Ilmenau, Germany; uwe.ritter@tu-ilmenau.de; 6 Helmholtz-Zentrum Hereon, 21502 Geesthacht, Germany

**Keywords:** COVID-19, TMPRSS2, C_60_ fullerene, binding sites, computer simulation

## Abstract

The recent global spread of the SARS-CoV-2 pathogen, which causes COVID-19, and its rapid mutation, requires the fast development of effective preventive and treatment measures. According to WHO reports, over 778 million confirmed cases of COVID-19 have been reported, including approximately 7 million deaths. The androgen-regulated cell-surface serine protease TMPRSS2 interacts with the SARS-CoV-2 spike protein. Therefore, directly inhibiting TMPRSS2 will negatively impact the activation of coronaviruses and, consequently, disease progression. That is why TMPRSS2 is a very important target in current drug discovery. On the other hand, it is known that C_60_ fullerene (a nearly spherical molecule consisting of 60 carbon atoms) exhibits activity against various protein targets. Here, for the first time, the potential binding of C_60_ fullerene with TMPRSS2 was investigated using different computer simulation methods, including p2Rank, PCA, gmx_MMPBSA analysis, molecular docking, and molecular dynamics simulations. As a result, four potential binding pockets on the TMPRSS2 surface that could interact with C_60_ fullerene were identified. Among all “C_60_ fullerene-TMPRSS2” complexes, one was selected as the most promising binding site based on the results of computational modeling evaluations. This opens up the prospect of creating new anticoronavirus drugs based on these carbon nanoparticles.

## 1. Introduction

First identified in Wuhan, China, COVID-19 rapidly spread around the world. Triggered by the SARS-CoV-2 pathogen, this pandemic has developed into an unprecedented international health emergency [1,2,3,4]. As of today, COVID-19 has resulted in millions of cases, and this number continues to increase [4,5,6,7,8], because no effective treatment is available. SARS-CoV-2 is part of the *Coronaviridae* family, one of the largest viral families, which comprises more than 54 species [9,10,11,12,13]. It is a member of the *Betacoronavirus* genus and is highly similar to the previous SARS-CoV, which emerged in 2002. The sequence similarity between those two viruses is, in some domains, near or more than 80% [14,15,16,17,18,19,20,21]. According to the latest studies for both SARS-CoV and SARS-CoV-2, two protein targets are highly interesting: ACE2 (angiotensin-converting enzyme 2) and TMPRSS2 (transmembrane protease serine type 2) on the host cell surface [22,23,24,25,26,27,28]. The ACE2 protein interacts with the spike protein of coronavirus. This is followed by activation of TMPRSS2, which cleaves the S1/S2 and S2’ sites to initiate fusion of the host cell membrane and the coronavirus envelope [29]. These two protein targets are present in different cells of various human organs, including the kidney, liver, heart, and others [30,31,32,33,34]. That is why we can conclude that, for example, TMPRSS2 is a crucial target for virus entry and spread in the body [28,32,35,36,37,38].

Since TMPRSS2 is less important compared to ACE2 in the human body’s homeostasis, inhibiting it will not have any impact on vital processes in the body [39]. However, TMPRSS2 plays a critical role in activating the SARS-CoV-2 S protein’s activity [40,41]. Therefore, by binding to TMPRSS2, it is possible to prevent the fusion of the SARS-CoV-2 envelope with the cell membrane. Another advantage of this protein is that, as a human protease, targeting it therapeutically lowers the risk of resistance development, unlike targeting viral proteins [42,43,44,45,46]. On this basis, we can consider TMPRSS2 to be one of the most promising anti-SARS-CoV-2 therapeutic targets [47,48]. Therefore, understanding the molecular mechanisms of TMPRSS2’s interaction with viral proteins and identifying novel inhibitors could contribute to the development of effective therapeutic strategies against SARS-CoV-2 and potential future coronavirus outbreaks.

Modern biomedical technologies, which widely use nanoparticles, can contribute to solving many clinical problems, including those that arose as a result of the COVID-19 pandemic [49,50]. That is why nanosized, almost spherical, and biocompatible C_60_ fullerene [51,52] is of practical interest, due to its inherent “specific” antiviral activity [53,54]. In addition, C_60_ fullerene is a unique molecular scaffold that is capable of modulating the functions of different proteins [55]. It has been shown that a chemically modified C_60_ molecule sterically blocks the lipophilic channel of the HIV-1 and HIV-2 proteases [56,57]. It is an effective inhibitor of the NS5B polymerase and NS3/4A protease of the hepatitis C virus [58,59]. In a model study [60], the ability of C_60_ fullerene to block the protein targets 3CLpro (chymotrypsin-like protease) and RdRp (RNA-dependent RNA polymerase) of the SARS-CoV-2 and thus to inhibit its functional activity was demonstrated. Finally, for the first time, the anticoronavirus activity of water-soluble C_60_ fullerenes was tested in an in vitro system [61]: as a model, being apathogenic for human coronavirus, a transmissible gastroenteritis virus of swine (TGEV), adapted to the BHK-21 cell culture (kidney cells of a newborn Syrian hamster), was used. The water-soluble C_60_ fullerenes at the maximum allowable cytotoxic concentration of 37.5 μg/mL reduced the titer of TGEV coronavirus infectious activity by a value of 2.00 TCID_50_/mL. Moreover, recently, an in vitro study was reported, revealing the potent anti-SARS-CoV-2 activity of water-soluble C_60_ fullerene derivatives with pendant carboxylic groups: time-of-addition analysis and molecular docking results indicated that the viral protease and/or the spike protein are the most probable targets [62].

Here, for the first time, we have conducted an analysis of the TMPRSS2 protein structure and its potential to interact with C_60_ fullerene utilizing computational methods. For this purpose, a detailed surface analysis of TMPRSS2 was performed. As a result, four binding pockets were selected and analyzed. Then, C_60_ fullerene was docked into the selected binding pockets 1–3; the 4th binding pocket was rejected, since it is flat and small. Subsequently, molecular dynamics (MD) simulations were performed using the docking results. The obtained MD trajectories were then subjected to gmx_MMPBSA (molecular mechanics Poisson–Boltzmann surface area) investigation. As a final result, one of the four binding pockets was selected as a promising binding pocket for C_60_ fullerene interaction on the TMPRSS2 protein surface.

## 2. Results

### 2.1. TMPRSS2 Binding Pocket Analysis

**Figure 1:**
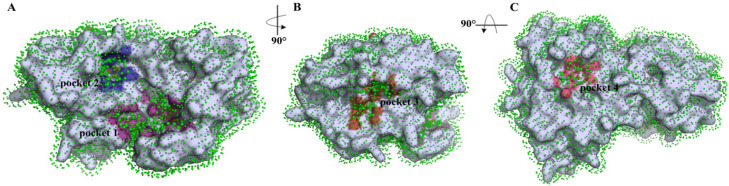
Ligand binding site visualization predicted by p2Rank. Protein (in gray) is covered by a layer of points (in green) lying on the SAS (solvent accessible surface) of the protein. Each point represents its local chemical neighborhood. Visualization is based on a PyMOL session generated by p2Rank. Binding pocket 1—magenta, 2—blue, 3—brown, 4—pink.

In accordance with the p2Rank analysis of TMPRSS2, four binding pockets were selected (Figure 1). The first binding pocket is located at the boundary between the SP (trypsin-like serine peptidase) and SRCR (scavenger receptor cysteine-rich) domains (Figure 1A). In contrast, binding pocket 2 is located within the SP domain only (Figure 1A). In the case of binding pocket 3, this one actually refers to the catalytic binding site, based on data from the literature [63] (Figure 1B). Finally, binding pocket 4 is situated on the opposite side of the SP domain compared to the other selected pockets (Figure 1C).

All the detected pockets are more or less small, which is actually optimal for C_60_ fullerene. Pockets 1–3 are characterized by an irregular shape with a slight indentation. Contrary to them, pocket 4 is flat. The analyzed pockets contain the following amino acids: pocket 1—polar: Ser 121, Tyr 161, Asn 336, Asn 344, Thr 481; non-polar: Ile 118, Pro 129, Trp 132, Trp 168, Pro 335, Ala 423, Val 479, Phe 480, Trp 482; charged: Glu 119, Arg 147, Arg 150, His 334, Arg 486; pocket 2—polar: Gln 374, Thr 407, Asn 476; non-polar: Met 372, Leu 373, Pro 375, Leu 404, Ile 405, Met 424, Ile 425, Cys 426, Ile 456, Met 478; charged: Glu 406; pocket 3—polar: Ser 436, Gln 438, Ser 441, Thr 459; non-polar: Val 280, Cys 281, Leu 302, Cys 437, Gly 439, Trp 461, Gly 462, Gly 464, Cys 465; charged: His 296, Glu 389; pocket 4—polar: Asn 247; non-polar: Ala 266, Trp 267, Trp 380, Trp 453; charged: Glu 260, Lys 401. Also, in p2Rank, we have the ability to characterize their internal parameter sas_points (a value often associated with Solvent Accessible Surface (SAS) Area). Through this, it is possible to evaluate how accessible a region is to solvent molecules [64,65]. According to p2Rank, pockets 1–4 contain 108, 40, 68, and 25 sas_points, respectively.

So, binding pocket 1 is the most solvent-exposed one (sas_points 108) and contains a mix of hydrophobic and polar residues. This pocket has a high potential to create different types of stacking interactions, since it comprises multiple residues like Arg, His, Trp, and Tyr. Binding pocket 2, located near binding pocket 1, is more hydrophobic, but the big advantage here is that this pocket is buried. Thus, C_60_ fullerene can entirely fill that pocket. Furthermore, it contains a few residues capable of forming stacking interactions with C_60_ fullerene, namely Met 372, Met 424, Cys 426, and Met 478. Binding pocket 3, like the previous one, is buried. Combined with its volume and the presence of amino acids that can create stacking interactions (Cys 281, His 296, Cys 437, Trp 461, and Cys 465), this pocket is a good candidate for targeting. Finally, because binding pocket 4 is very small and flat, despite the fact that it contains many residues that can create stacking interactions (Trp 267, Trp 380, Lys 401, and Trp 453), we have decided to exclude it from further investigation.

So, within the identified binding pockets, pockets 1, 2, and 3 appear most promising for C_60_ fullerene targeting because of their shape, residue environment, and volume. Pocket 4 was removed from the investigation.

### 2.2. “C_60_ Fullerene-TMPRSS2” Interaction Analysis

**Figure 2:**
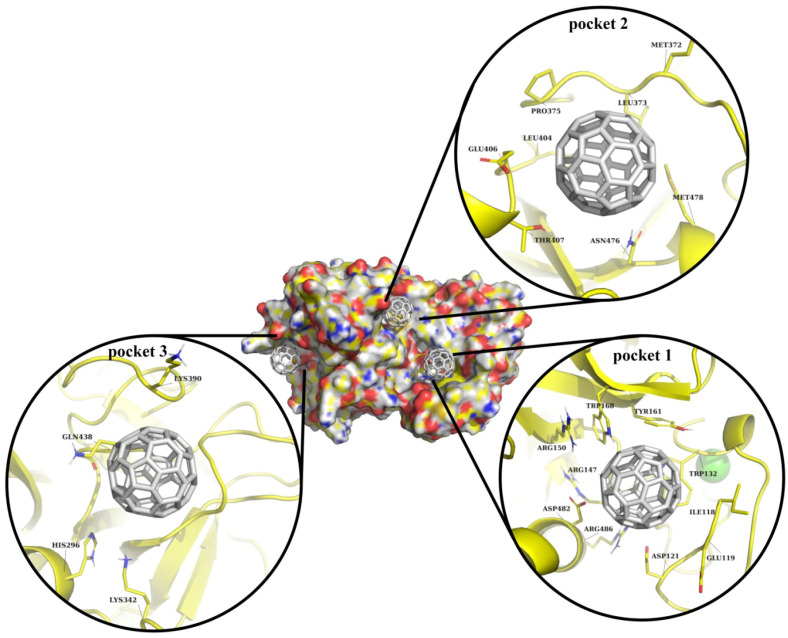
Visualization of C_60_ fullerene docking into predicted binding pockets, shown using surface and cartoon representations. In all cases, TMPRSS2 is depicted in yellow and C_60_ fullerene in gray.

Here, our focus was on the investigation of the interaction between C_60_ fullerene and selected binding pockets (Figure 2). In binding pocket 1, C_60_ fullerene is tightly bound between the loop and the α-helical region. The C_60_ fullerene fills only part of binding pocket 1, creating π/π-stacking interactions with Trp 168, Tyr 161, and Trp 132, and cation-π interactions with Arg147, Arg150, and Arg486. It seems that such diverse stacking interactions should stabilize the “C_60_ fullerene–TMPRSS2” complex and influence the investigated protein functions. Binding pocket 2 is mostly hydrophobic, so it is fair to assume that binding inside that pocket will be less favorable compared to binding pocket 1. Here, C_60_ fullerene is flanked by polar residues including Thr 407, Asn 476, and Glu 406. On the other hand, the binding pocket comprises two Met amino acids that could create stacking interactions with C_60_ fullerene, thus fixing it in this binding pocket. However, according to the data from the docking simulation, in its current conformation, Met 372 is a bit too far from C_60_ fullerene to create stacking interactions. However, considering that Met 372 is flexible, it is still possible. In the case of binding pocket 3, C_60_ fullerene lies above the catalytic binding pocket, shielding it from interaction with any other chemical structures. This pocket is the smallest, including a smaller amount of residues, but almost all of them are charged and are polar amino acids, such as His 296, Lys 342, Lys 390, and Gln 438. All those residues are capable of forming cation-π or stacking interactions with C_60_ fullerene.

In our opinion, binding with any of those pockets could potentially impact TMPRSS2 functionality. It looks like, based on the molecular docking simulation, pocket 1 potentially represents the most promising TMPRSS2 site for C_60_ fullerene binding. Binding pockets 2 and 3 seem to be less attractive, since the first one cannot form a sufficient number of interactions with C_60_ fullerene, and the second one is small. Nevertheless, all this data should be evaluated using more accurate methods, e.g., MD simulation.

### 2.3. “C_60_ Fullerene-TMPRSS2” Stability Analysis

**Figure 3:**
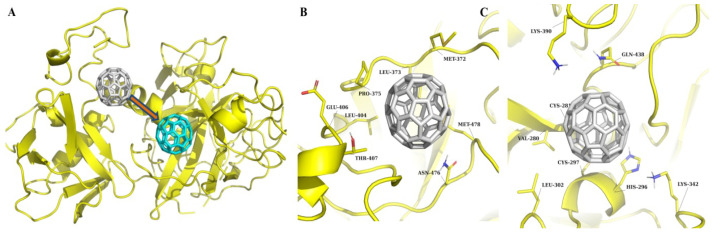
MD simulation results of TMPRSS2 in complex with C_60_ fullerene. (**A**) Binding pocket 1: The initial position of C_60_ fullerene is shown in gray, and its position after simulation is shown in cyan. (**B**) Interaction at binding pocket 2. (**C**) Interaction at binding pocket 3. TMPRSS2 is shown in yellow in all panels, and C_60_ fullerene in gray, except for the post-simulation pose in A (cyan).

**Figure 4:**
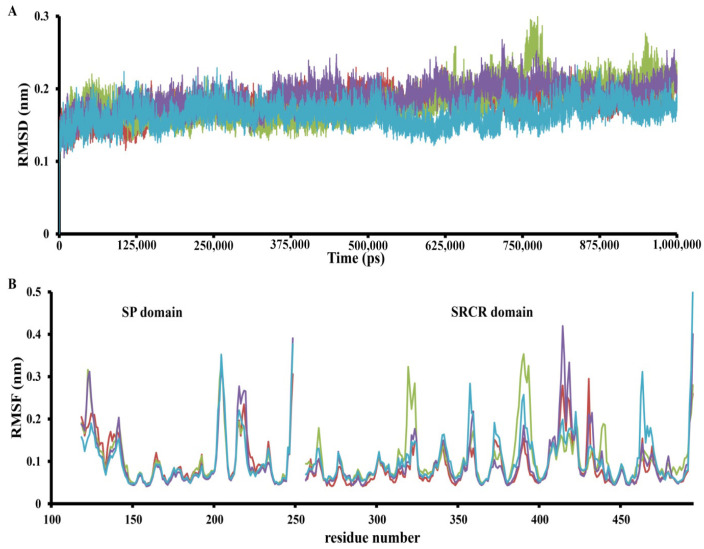
The RMSD (**A**) trajectory and RMSF (**B**) MD trajectory profile of apo TMPRSS2 (red), C_60_ fullerene interaction in binding pocket 1 (green), binding pocket 2 (violet), and binding pocket 3 (cyan).

In order to achieve greater accuracy, a 1000 ns MD simulation was performed (Figure 3 and Figure 4). The simulation results showed that some predicted complexes remained stable throughout the simulation. The RMSD (root-mean-square deviation) movement in all the complexes stayed within the 1–2 Å range (Figure 4). Interestingly, in some cases, complexes completely lost all interactions in the binding pocket; in others, they established new, potentially stronger interactions.

The results of the TMPRSS2 MD simulation are presented in Figure 3 and Figure 4. The “C_60_ fullerene-binding pocket 1” complex showed signs of instability, rapidly losing crucial interactions with TMPRSS2 just after the start of the MD simulation. Here, C_60_ fullerene eventually completely relocated to the predicted binding pocket 2. As for the interaction insights from binding pocket 2, it was very stable. The C_60_ fullerene was immersed deeper inside the binding pocket, shifting by 2.6 Å. That displacement caused changes in the pocket conformation and interaction with C_60_ fullerene. From the close-up view, it was possible to determine different types of interactions, such as hydrophobic/steric contacts and van der Waals forces, which participate in complex stabilization. Interestingly, this effect was mostly achieved by hydrophobic/steric interactions with C_60_ fullerene, including its contacts with Leu 373, Pro 375, Thr 407, Glu 406, Leu 404, and Asn 476. In terms of stacking, there were stable interactions with Met 478 and an additional one with Met 372. Met 372 is located at the edge of the pocket and can move freely. As a result, Met 372 changed its position and covered C_60_ fullerene, holding it in place. The C_60_ fullerene binding with pocket 3 was characterized by huge changes in the binding model. It was observed that C_60_ fullerene shifted by 3.2 to 7.8 Å inside the catalytic binding pocket, causing various residues to change. For example, Lys 390 and 342 dramatically changed their conformations and created cation-π interactions with C_60_ fullerene. In contrast, His 269 slightly changed its conformation and at the same time anchored C_60_ fullerene in the binding pocket through π-stacking interactions. The most intriguing aspect here is that, as a result of the MD simulation, C_60_ fullerene completely closed the catalytic binding pocket so no other binding agent could reach it. That would definitely have a significant impact on TMPRSS2 functionality.

Finally, the “C_60_ fullerene-TMPRSS2” complex was subjected to RMSF (root-mean-square fluctuation) analysis. Overall, the RMSF values remained below 0.4 nm for most residues, suggesting protein stability during the simulation (Figure 4). Nevertheless, for the SP and SRCR domains, several distinct dynamic regions could be observed. The SP domain displayed a few RMSF differences, with one region that behaved differently in each investigated model. This region corresponds to residues 225–235 and is interesting because it is not located near any binding pockets. So, that was probably a distance effect of C_60_ fullerene binding in different models. However, this is just an assumption and requires deeper investigation. In contrast, the SRCR domain contains multiple parts that demonstrated different fluctuations depending on the binding model: these are residues 310–320, 350–360, 385–405, 410–445, and 460–470.

So, according to the obtained results, C_60_ fullerene showed dynamic behavior, relocating from binding pocket 1 to binding pocket 2. That is a surprising result, since binding pocket 1 comprises many residues able to create stacking interactions with C_60_ fullerene. In contrast, binding pocket 2 contains just two Met residues, and most of the other residues are able to create steric/hydrophobic interactions with C_60_ fullerene. As for binding pocket 3, the C_60_ fullerene inserted more deeply into the catalytic site and completely blocked one. According to the fluctuation analysis, TMPRSS2 is stable, with some fluctuations in both the SP and SRCR domains.

### 2.4. PCA Study

**Figure 5:**
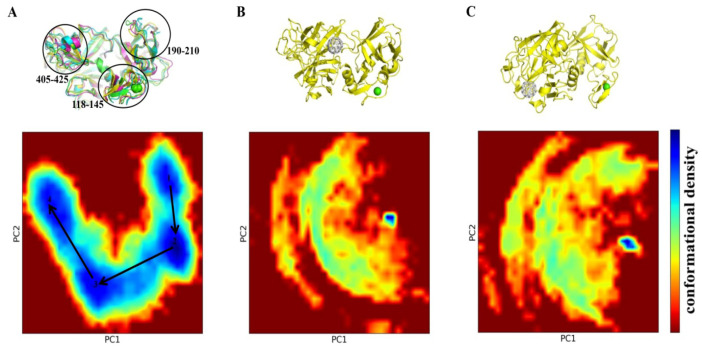
PCA of TMPRSS2 dynamics and its interaction with C_60_ fullerene. (**A**) PCA of TMPRSS2 apo form reveals major conformational changes in regions 118–145, 190–210, and 405–425. With this case as the central example, the following snapshots were selected 1—18,950 (green), 2—350,400 (cyan), 3—708,950 (violet), 4—864,450 (yellow). (**B**) PCA plot and structure of TMPRSS2 in complex with C_60_ fullerene at binding pocket 2. (**C**) PCA plot and structure of TMPRSS2 in complex with C_60_ at binding pocket 3. Conformational landscapes are shown as 2D density plots along the first two principal components (PC1 and PC2), with the color scale indicating conformational density (blue = high density, red = low density). TMPRSS2 is shown in yellow, and C_60_ fullerene in gray.

The application of principal component analysis (PCA) suggests (Figure 5) that TMPRSS2 alone and in complex with C_60_ fullerene have noticeably different conformational behaviors. The TMPRSS2 apo form can assume a significant number of different conformations (Figure 5A). It indicates a significant structural flexibility of the TMPRSS2 structure. However, it should be noted that the main structure of the apo TMPRSS2 remained relatively rigid during the simulations; the conformational variations were mainly observed in the loop regions. In contrast, the “C_60_ fullerene-TMPRSS2” complexes demonstrated a dramatic reduction in conformational space, with only one small conformationally dense part (Figure 5B,C). In accordance with that, we can suggest that binding with C_60_ fullerene imposes structural constraints on TMPRSS2 and fully stabilizes only one specific conformation of the “C_60_ fullerene-TMPRSS2” complex.

### 2.5. Binding Affinity Analysis via MMPBSA

For a better understanding of the binding properties between TMPRSS2 and C_60_ fullerene, a binding energy analysis has been conducted (Table 1). The van der Waals (vdW) interactions have the most impact on the overall binding affinity, with values of −49.56 and −33.28 kcal/mol for pockets 2 and 3, respectively. Those values represent the hydrophobic nature of C_60_ fullerene, which interacts primarily through dispersion forces with non-polar residues inside the pocket.

In contrast, the electrostatic interaction (EE), polar solvation (PS), and non-polar solvation (NPS) energies have minimal or unfavorable contributions to complex formation in both binding pockets. So, the EE energy contributes almost nothing to binding (−0.09 and −0.04 kcal/mol), and that is actually expected, because C_60_ fullerene is neutral, and cannot form classical electrostatic interactions. Similarly, in the case of PS, it is unfavorable for both investigated binding pockets (12.30 and 9.41 kcal/mol), which indicates a significant potential binding penalty during the transfer of the hydrophobic C_60_ fullerene from solvent into the investigated binding pocket. And finally, the NPS represents only slight compensation compared to the two previously demonstrated energy types (−3.21 and −2.33 kcal/mol). As a result, the binding energy (TOTAL) is significantly more favorable for pocket 2 (−40.56 kcal/mol) compared with pocket 3 (−26.24 kcal/mol); it indicates that binding pocket 2 has better geometric and hydrophobic complementarity to the C_60_ fullerene surface.

Also, to estimate system efficiencies, a thermodynamic analysis has been performed (Table 2). For binding pocket 2, the enthalpic contribution (∆H) was more favorable (−40.56 kcal/mol) compared to binding pocket 3 (∆H is −26.23 kcal/mol). This indicates stronger interaction in the case of binding pocket 2. As a result, the final Gibbs free energy (∆G) is favorable for binding pocket 2 (−30.71 kcal/mol), despite ∆G (0.45 kcal/mol) for binding pocket 3 indicating very weak or no binding in this pocket with TMPRSS2.

Together, these results demonstrate that vdW interactions overwhelmingly determine the binding preference, and that only pocket 2 provides a sufficiently hydrophobic and shape-complementary environment for stable C_60_ fullerene association with TMPRSS2.

## 3. Discussion

Recent studies have pointed to TMPRSS2 as one of the key targets that facilitates SARS-CoV-2 entry to the host cells [28,32,35,36,37,38,66]. TMPRSS2 is reported as a multidomain serine protease consisting of an SRCR, trypsin-like SP domains, and a linker region between them [67,68]. The SP domain itself contains the only known well-characterized binding pocket, which includes the catalytic triad His–Asp–Ser [30,42,63,69]. This region, referred to as binding pocket 3, was also identified in our analysis. The obtained results have demonstrated that this pocket is relatively narrow, which explains why it typically interacts with elongated, aromatic ligands such as camostat and nafamostat [47,63,70]. However, our analysis also demonstrates that this pocket is enriched with polar and charged amino acid residues capable of creating π–π and cat-ion–π interactions (e.g., His 269 and Cys 465). So, binding pocket 3 is potentially able to interact with spherical structures like C_60_ fullerene.

In contrast, the remaining pockets have not been well described in the available literature. According to the available data [39,47,70,71], TMPRSS2 contains secondary pockets adjacent to and distinct from the catalytic cleft. However, many studies are focused on the catalytic binding pocket’s description and its different possible sub-pockets [39,70]. Contrary to this, the authors of [47,71] describe all potential high-affinity binding spots for chemicals (e.g., camostat and nafamostat). However, the binding pocket mapping in each publication is different. For example, the region that we define as pocket 2 is divided into two distinct sub-sites in their models (M-5F8T_site_1 and M-5F8T_site_4) [71]. Overall, previous studies have made only limited attempts to select binding pockets distinct from the catalytic one, and these efforts have primarily concentrated on essentially the same parts of the TMPRSS2 protein, that is, on the interface between the SRCR and SP domains and the SP domain, on the opposite side from the location of the catalytic binding pocket. Thus, our analysis systematically identifies and characterizes these additional binding pockets, expanding the current understanding of TMPRSS2’s ligand-binding landscape beyond the classical catalytic site.

The obtained MD simulation and docking results provide important insights into the interaction between C_60_ fullerene and TMPRSS2. Although docking initially suggested that the binding pocket 1 was one of the most favorable sites due to its presence of aromatic and charged residues, nevertheless, the long-timescale MD simulation revealed that those potential interactions with C_60_ fullerene are unstable. That is actually not surprising, because this region is located near flexible parts of the protein (Figure 5), making ligand binding in such a region often transient and unstable [72,73,74,75]. This actually explains why C_60_ fullerene migrates from pocket 1 into pocket 2 during the MD simulation. It is interesting that binding pocket 2 comprises fewer aromatic residues. However, it has a deeper pocket cavity and is located in a less flexible region of TMPRSS2. A similar picture is observed in the studies of [47,71], where camostat binds in the SRCR and SP domain interface, with a docking energy of −9.95 kcal/mol, but during the MD simulation it demonstrates conformational plasticity of this region. All those data suggest that binding pocket 1 initially appears favorable based on static docking, but dynamic fluctuations and solvent reduce its stability, and as a result, different potential binders lose their ability to interact with one. Therefore, we suggest that the C_60_ fullerene migration from binding pocket 1 to pocket 2 during MD simulation reflects a fundamental transition from an un-favorable state to a preferred, more stable complex, where the C_60_ fullerene is buried within a deeper, less mobile cavity.

The gmx_MMPBSA results demonstrate that the van der Waals forces are dominant in the complex formation between C_60_ fullerene and TMPRSS2 protein. This behavior is fully consistent with the intrinsic physicochemical properties of C_60_ fullerene, a neutral hydrophobic molecule that interacts mainly through dispersion forces [76,77]. In contrast, classical TMPRSS2 inhibitors such as camostat and nafamostat rely on a combination of electrostatic and hydrogen bonding with the TMPRSS2 catalytic binding pocket [47,70]. So, that is aligned with our results. Based on the gmx_MMPBSA analysis, it is highly unlikely that C_60_ fullerene would bind to the catalytic binding pocket. Rather, it interacts with the TMPRSS2 only via a non-classical binding mechanism, driven by hydrophobic and dispersion interactions, predominantly. And actually, binding pocket 2 provides a sufficient residue environment and geometric complementarity for C_60_ fullerene binding, whereas binding pocket 3 (catalytic pocket) is smaller and more polar, which limits its ability to stabilize the C_60_ fullerene. Overall, compared to the previously studied “TMPRSS2–ligand” systems, where polar interactions are dominant, our results reveal a distinct binding model that is based on pocket geometry and dispersion forces.

## 4. Materials and Methods

### 4.1. Hardware and Software

All the computational study was carried out on Ubuntu 24.1 (64-bit) operating systems equipped with 96 GB of RAM and 3 × 3.60 GHz AMD Ryzen™ 5 1600X Six-Core processors, except for the MD simulations. The MD simulations were performed using 3× Gigabyte GeForce RTX 3060 12228MB GPUs. For visualization, PyMOL (v3.1) [78] and Matplotlib (v3.10) [79] tools were used.

### 4.2. Selection of Protein Structure and Binding Site Determination

Based on the available data, the Protein Data Bank (PDB) [80] contains 30 structures. All the structures were retrieved and analyzed. Initially, all crystallographic water molecules and native ligands were removed from the protein structure. Next, the structure was refined by adding missing hydrogens, correcting amide protonation states, and repairing incomplete side chains using MolProbity 4.5.2 [81,82]. The system was further minimized using the CHARMM36 force field to remove all the strains and steric clashes inside the protein system [83,84,85]. Finally, taking into consideration structure integrity, resolution, host organism (human), and novelty, 9jd1 PDB ID [86] was selected for further investigation.

In the next stage, the binding pockets for C_60_ fullerene were defined by the p2Rank 2.5 [68] software tool. p2Rank evenly spread the points on the protein’s SAS. Each point served as the center of its own spherical region, which together may represent possible binding pockets for different binders. To predict potential binding pockets, p2Rank ran the following steps:

1. Generation of spaced points on a protein SAS, based on a fast numerical algorithm [87] implemented in the CDK library [88,89].
2. Calculation of the location of points on the protein surface.
3. Prediction of ligand ability of SAS points employing a Random Forest approach [90,91].
4. Clustering of all the points to create potential binding pockets (the cut-off distance 3 Å).
5. Ordering predicted binding pockets by the ligand ability score of the points they contain (sum of squared ligand ability scores of all points in the cluster).

### 4.3. Molecular Docking Study

Both C_60_ fullerene and TMPRSS2 were prepared before the molecular docking simulation. In the case of TMPRSS2, all the preparation data were retrieved from the previous stage (preparation based on MolProbity). The C_60_ fullerene coordinates were downloaded from the MSU Fullerene Database. Then, OpenBabel 3.1.0 software was applied to prepare the C_60_ fullerene for the molecular docking simulation [92]. Specifically, the protonation and geometry of the structure were checked to ensure proper chemical integrity before the simulation. The molecular docking simulations were performed utilizing SwissDock 2024 [93], using cavities 2.0 (AC) algorithm (a detailed description of the docking algorithm is provided in [94,95]). The binding pockets were defined in accordance with p2Rank results.

Prior to running the molecular docking simulation, the central amino acids in each binding pocket were indicated. For each pocket, residues located within a 6.0 Å sphere of the central amino acid were considered part of the binding pocket. Specifically, pocket 1 was centered on Arg 147, pocket 2 on Leu 404, and pocket 3 on Gln 438. In each docking case, the 10 best complexes were selected for analysis stages. In the end, the molecular docking results were estimated using the docking score and visual inspection of all the obtained poses.

### 4.4. MD Simulation Strategy

To evaluate the stability and key interactions of the obtained complexes after molecular docking, MD simulation was carried out. The calculations were performed using GROMACS 2024.2 [96] with the CHARMM36 force field [83,84,85]. The TMPRSS2 protein was protonated according to the built-in function in GROMACS 2024.2—“-ignh”. The topology for C_60_ fullerene was generated by SwissParam 2023 [97,98]. The best complexes obtained after molecular docking were used for MD simulation. Each system was placed at the center of a periodic cubic box, which was then filled with TIP3P water molecules. A minimum 10 Å distance was maintained between the nearest atom of the complex and the edge of the simulation box so that the complex could be fully immersed in water and rotate freely. Then, to neutralize the system and mimic the cellular environment (pH = 7), Na^+^ and Cl^−^ ions were added to bring the ionic concentration to 150 mM. Here, the solvent molecules were randomly replaced with monoatomic ions. Next, the obtained complex was energy minimized, which also relieved any steric clashes. The system was relaxed by applying the steepest descent algorithm (the maximum number of steps was 50,000). Then the equilibration was computed in two stages: NVT (constant volume-temperature simulation) was first equilibrated for 5 ns, followed by NPT (constant pressure-temperature simulation) equilibration for 5 ns. After that, we launched the MD simulation for 1000 ns. All calculations were performed at a temperature of 300 K and at constant atmospheric pressure.

### 4.5. PCA Study

PCA has been applied to reveal high-amplitude concerted motions within the structure of the TMPRSS2 protein [99,100,101]. The simulations were carried out utilizing eigenvectors derived from the mass-weighted covariance matrix of RNA atomic fluctuations. To generate the covariance matrix, the built-in GROMACS 2024.2 function ‘gmx covar’ was used. Then, we calculated eigenvectors and eigenvalues to define the dimensionality of the essential subspace. Note that a small subset of eigenvectors (<10) characterizes 90% of all motions, revealing the main motions occurring within an atomic system. Cosine content is used as a measure of principal components [102,103]. If the cosine content is close to 1, it indicates significant movement within the TMPRSS2 molecule and renders it unsuitable for PCA. However, most snapshots captured during MD simulation exhibit cosine values close to 0.2, with some approaching 0.5, making them suitable for PCA. Thus, according to the above, by using the ‘gmx analyse’ utility, the FEL (free energy landscape) was constructed utilizing cosine contents <0.2 of the first two projection eigenvectors (defined as PC1 and PC2, respectively). The most prevalent and energetically favorable structures extracted from the FEL’s minimum energy basins were then utilized for subsequent analyses.

### 4.6. Binding Energy Calculation

The MM/PBSA molecular mechanics [104,105,106,107] was executed to estimate “C_60_ fullerene-TMPRSS2” complex binding energy using the gmx_MMPBSA v1.6.3 [106] software tool. This is an Amber-based tool [108] that calculates binding energy (∆E_TOTAL_) based on the following equation

∆E_TOTAL_ = E_vdW_ + E_EE_ + E_PS_ + E_NPS_,

where E_vdW_—van der Waals energy; E_EE_—electrostatic energy; E_PS_ and E_NPS_—polar and non-polar solvation energy, respectively, and also the Gibbs free energy (∆G):

∆G = ∆H − TΔS.

Here ∆H—enthalpy and T∆S—entropic term.

## 5. Conclusions

The TMPRSS2 is essential for SARS-CoV-2 activation and entry. That is why this protein represents an important therapeutic target. On the other hand, nanostructures like C_60_ fullerene have been widely discussed as unconventional molecular scaffolds that are capable of modulating the functions of different proteins. So, understanding whether C_60_ fullerene is able to interact with TMPRSS2 is significant in the context of a potential alternative antiviral therapy design.

In this study, a comprehensive computational investigation to evaluate the C_60_ fullerene’s ability to interact with TMPRSS2 was conducted. According to the p2Rank investigation, four binding pockets were identified. Among them, pockets 1–3 have the most promising shape and amino acid residue environment for C_60_ fullerene targeting, while pocket 4 was unsuitable due to its flat architecture. According to subsequent simulations, the C_60_ fullerene interaction with binding pocket 1 is not stable, and based on thermodynamic analysis, binding in pocket 3 is very weak or not possible at all. So, among all the evaluated binding pockets, pocket 2 seems to be the most promising one for binding with C_60_ fullerene.

Furthermore, further simulations have shown that C_60_ fullerene noticeably restricts the conformational dynamics of TMPRSS2, while the unbound protein has a broader conformational landscape. This suggests that C_60_ fullerene may fix specific structural states of TMPRSS2, and such binding can potentially affect its biological functions, in particular to block interactions with SARS-CoV-2.

## Figures and Tables

**Table 1:** The calculated energy contributions of TMPRSS2 binding with C_60_ fullerene in pockets 2 and 3 (in kcal/mol).

Pocket	vdW	EE	PS	NPS	Total
2	−49.56	−0.09	12.30	−3.21	−40.56
3	−33.28	−0.04	9.41	−2.33	−26.24

**Table 2:** The calculated thermodynamic parameters of C_60_ fullerene binding in TMPRSS2 selected binding pockets 2 and 3 (in kcal/mol).

Pocket	∆H	T∆S	∆G
2	−40.56	−9.85	−30.71
3	−26.23	−26.68	0.45

## Data Availability

The data presented in this study are available on request from the corresponding author.
